# Platelet-derived mediators in hospitalized COVID-19 patients and associations to respiratory failure, ICU admittance and 60-day mortality

**DOI:** 10.3389/fcvm.2026.1685861

**Published:** 2026-02-25

**Authors:** Kari Otterdal, Thor Ueland, Jan Cato Holter, Mari Kaarbø, Ylva Schanke, Beathe Kiland Granerud, Tuva B. Dahl, Anders Tveita, Anders Benjamin Kildal, Lars Heggelund, Anne Hege Aamodt, Aleksander Rygh Holten, Kristian Tonby, Pål Aukrust, Anne Ma Dyrhol-Riise, Bente Halvorsen, Annika E. Michelsen

**Affiliations:** 1 Research Institute of Internal Medicine, Oslo University Hospital Rikshospitalet, Oslo, Norway; 2 Institute of Clinical Medicine, Faculty of Medicine, University of Oslo, Oslo, Norway; 3 Thrombosis Research Center (TREC), Division of Internal Medicine, University Hospital of North Norway, Tromsø, Norway; 4 Department of Microbiology, Oslo University Hospital, Oslo, Norway; 5 Section of Clinical Immunology and Infectious Diseases, Oslo University Hospital, Oslo, Norway; 6 Department of Internal Medicine, Bærum Hospital, Vestre Viken Hospital Trust, Gjettum, Norway; 7 Department of Anesthesiology and Intensive Care, University Hospital of North Norway, Tromsø, Norway; 8 Department of Clinical Medicine, Faculty of Health Science, UIT-The Arctic University of Norway, Tromsø, Norway; 9 Department of Internal Medicine, Drammen Hospital, Vestre Viken Hospital Trust, Drammen, Norway; 10 Department of Clinical Science, Faculty of Medicine, University of Bergen, Bergen, Norway; 11 Department of Neurology, Oslo University Hospital, Oslo, Norway; 12 Department of Neuromedicine and Movement Science, the Norwegian University of Science and Technology, Trondheim, Norway; 13 Institute of Population Health, Faculty of Health and Life Sciences, University of Liverpool, Liverpool, United Kingdom; 14 Department of Acute Medicine, Oslo University Hospital, Oslo, Norway; 15 Department of Infectious Diseases, Oslo University Hospital, Oslo, Norway

**Keywords:** COVID-19, inflammation, platelets, SARS-CoV-2, thrombosis

## Abstract

**Background:**

Platelet activation is documented in COVID-19, but data on platelet-derived mediators are scarce.

**Objective:**

To examine the levels of various platelet-derived mediators in relation to adverse outcomes defined as the need for treatment at intensive care unit (ICU) and/or respiratory failure (RF) and 60-day total mortality in hospitalized COVID-19 patients.

**Methods:**

Plasma levels of RANTES/CCL5, PF4/CXCL4, ENA78/CXCL5, NAP-2/CXCL7, SDF-1/CXCL12, P-selectin, soluble CD40 ligand (sCD40L) and vascular endothelial cell growth factor A (VEGF-A) were measured in 245 hospitalized COVID-19 patients and in a subpopulation of the patients, also at three, six and 12 months after hospitalization.

**Results:**

Our main findings were: (i) High levels of P-selectin was associated with ICU/RF, while low levels of PF4/CXCL4, ENA-78/CXCL5 and NAP-2/CXCL7 were associated with 60-days mortality. (ii) Most of the mediators were normalized after hospitalization, but plasma levels of sCD40L, ENA-78/CXCL7 and VEGF-A were markedly elevated compared to healthy controls for up to 12 months after hospitalization. (iii) *In vitro*, inactivated SARS-CoV-2 induced a dose-dependent release of NAP-2, P-selectin, RANTES, sCD40L and VEGF-A from isolated platelets.

**Conclusion:**

Our findings underscore the role of platelet-derived inflammatory mediators in the pathogenesis of COVID-19, potentially involving direct effects of SARS-CoV-2. The study also points to a persistent platelet activation following hospitalization.

## Introduction

Platelets are involved in COVID-19, predisposing to arterial and venous thromboses, in particular in critically ill patients (1, 2). Platelet activation during COVID-19 may be part of a general immune activation and inflammation (3, 4), but may also be a direct effect of SARS-CoV-2. If platelets are infected by SARS-CoV-2 directly through angiotensin-converting enzyme 2 (ACE2) or not is still debated, and also other receptors for SARS-CoV-2 have been proposed (5, 6).

In COVID-19 patients, platelet counts, size, and maturity and increased membrane expression of P-selectin and CD40 ligand (CD40L) have been related to disease severity (7). It has also been shown that platelets from COVID-19 patients, in response to thrombin, exhibit an increased capacity to release soluble CD40L (sCD40L) and interleukin (IL)-1β, which correspond to disease severity (2). Moreover, plasma levels of P-selectin have been suggested as an early marker of thromboembolism in COVID-19 underscoring the role of platelets in COVID-19 complications (8). Furthermore, decreased platelet counts, reflecting enhanced platelet consumption or dysregulated production, has widely been shown to be associated with adverse outcome as well as a dysregulated immune response in COVID-19 disease (9–11), but the importance of the different platelet-derived mediators in relation to outcome is still unclear. Moreover, persistent platelet activation is reported in relation to pulmonary impairment (12, 13), potentially involving the interaction between platelets and neutrophils (14). However, the levels of the different platelet-derived mediators following the acute infection are still unclear.

To further elucidate the role of platelet activation in COVID-19, we examined the levels of various platelet-derived mediators in relation to the need for admission to the intensive care unit (ICU) and/or respiratory failure (RF) *and* 60-day total mortality in hospitalized COVID-19 patients, as well as the levels of these mediators following the acute infection for up to 12 months post-discharge. To demonstrate that platelets are a likely source of these biomarkers, we performed additional *in vitro* experiments exposing platelets to inactivated SARS-CoV-2.

## Materials and methods

### The study cohort

The patient cohort (*n* = 294), the Norwegian SARS-CoV-2 study (NCT04381819), was an observational study of hospitalized COVID-19 patients admitted to five Norwegian hospitals, conducted as part of the International Severe Acute Respiratory and Emerging Infection Consortium (ISARIC) WHO Clinical Characterization Protocol study (15). In this study, plasma samples were available from 290 patients with 245 patients during hospitalization and 176 patients had a follow-up sample at either three (*n* = 151), six (*n* = 68) and/or 12 months (*n* = 67). Of the follow-up samples, 45 patients lacked samples during hospitalization.

Patients ≥18 years who were admitted to the hospital with polymerase chain reaction (PCR)-confirmed SARS-CoV-2 infection were eligible for inclusion. We collected three serial blood samples throughout the first ten days of hospitalization: within 48 h of admission, on days three to five and on days seven to ten. Additionally, in a subgroup of patients, we collected blood samples at three (*n* = 151), six (*n* = 68), and 12 (*n* = 67) months following hospital discharge.

The patients were enrolled from March 2020 to September 2021, encompassing the initial three waves of the COVID-19 pandemic in Norway; wave one, March 8, 2020, to July 31, 2020; wave two, August 1, 2020, to February 17, 2021; and wave three, February 18, 2021, to July 31, 2021. By February 2021, the alpha variant was predominant, later replaced by the delta variant in July 2021.

### Ethics

The study was approved by the Regional Committees for Medical Research Ethics South East Norway (approval no. 106624; 13.02.2020) and performed according to the Declaration of Helsinki. Healthy controls used for the *in vitro* experiments were recruited through the CovaxHEAD study (approval no. 351097). All participants gave informed consent prior to inclusion, either directly or through a legally authorized representative.

### Outcomes

The pre-defined outcomes in this study were to examine levels of platelet-derived molecules in relation to disease severity: (1) RF, defined as PaO_2_/FiO_2_ (P/F) ratio <26.6 kPa (<200 mmHg) and/or the requirement for admission to ICU during hospitalization, and (2) 60-day post-admission all-cause mortality. In addition, we evaluated (3) the levels of these markers at three-, six-, and 12-months following hospital discharge.

### Blood sampling protocol

Peripheral venous blood was drawn into pyrogen-free blood collection tubes with ethylenediamine tetraacetic acid (EDTA) as anticoagulant, immediately immersed in melting ice, and centrifuged at 2,500 g for 20 min at 4°C within 60 min to obtain platelet-poor plasma. The plasma was aliquoted and stored at −80°C until analysis and thawed <3 times.

### Biochemical measurements

Soluble levels in patients’ plasma or in samples from *in vitro* experiments of *R*egulated on *A*ctivation, *N*ormal *T*-cell *E*xpressed and *S*ecreted (RANTES/CCL5), P-selectin, soluble CD40 ligand (sCD40L), stromal cell-derived factor 1 (SDF-1/CXCL12), detecting both the α and β isoforms, platelet factor 4 (PF4/CXCL4), epithelial neutrophil-activating protein 78 (ENA-78/CXCL5), chemoattractant neutrophil-activating peptide-2 (NAP-2/CXCL7) and vascular endothelial growth factor (VEGF)-A were measured in duplicates by enzyme immunoassay (EIA) using commercially available antibodies (R&D Systems, Minneapolis, MN), except for VEGF-A where antibodies were obtained from PeproTech (Cranbury, NJ). The EIAs were analyzed in a 384-format using a combination of a SELMA pipetting robot and a BioTek (Santa Clara, CA) dispenser/washer. Absorption was read at 450 nm with wavelength correction set to 540 nm using an EIA plate reader (BioTek). The intra-assay coefficient of variation based on data from our laboratory was <10%.

For reference, the actual markers were also analyzed in plasma from 29 age- and sex-matched healthy controls (mean age ± SD: 55 ± 12 years) using the same blood sampling protocol and storage conditions as for the patients.

Routine laboratory variables [e.g., C-reactive protein (CRP) and platelet, total leukocyte, neutrophil, lymphocyte, and monocyte counts, and creatinine/estimated glomerulus filtration rate (eGFR)] were measured at the biochemical laboratories at the participating hospitals.

### Preparation of SARS-CoV-2 virus

Alpha SARS-CoV-2 variant (SARS-CoV-2 strain 2019-nCoV/Italy-INMI1), originally obtained from the European Virus Archive, was used. High titer virus stocks were made by infecting confluent Vero E6 cells (Cat. no. CRL-1586; American Type Culture Collection Gaithersburg, MD) with 0.01 multiplicity of infection (MOI) SARS-CoV-2 in Dulbecco's Modified Eagle Medium (Cat. no. D6429; Merck Rahway, NJ) with 2% fetal calf serum (FCS; Cat. no. F7524, Merck) 1% l-glutamine (Merck), and 1% Pen/Strep (Thermo Fischer Waltham, MA) in 175 cm^2^ cell culture flasks. The cells were monitored daily for cytopathic effect under an inverted microscope. Upon nearly complete cell lysis (96 h post-infection), the viral supernatant was harvested, and cleared of cellular debris by centrifugation (500 g for 10 min) at 4°C. The clarified supernatant was passed through a 0.40 µm cell strainer (Corning, Corning, NY), aliquoted and stored at −80°C. Virus titer was determined by calculating the 50% tissue culture infectious dose. The virus was heat-inactivated at 65°C for 30 min (16).

### Platelet preparation and *in vitro* stimulation

Venous blood from healthy donors (*n* = 6) was drawn without stasis into 3.8% sodium citrate containing vacutainers (1/9 v/v) and centrifuged at 300 g for 8 min. Platelet-rich plasma (PRP) was collected, platelets were counted and thereafter diluted in RPMI (Cat. No. L0496, Biowest, Nuaillé, France) with 2% heat inactivated fetal calf serum (FCS; Cat. no. S181H, Biowest) obtaining 3.333 × 10^5^ platelets/mL. One million platelets in sterile polypropylene cryotubes (Nalgene NUNC, Rochester, NY) were incubated with virus suspension [0.1, 0.25, 0.5, or 1 multiplicity of infection (MOI)] for 4 h on rotation at 37°C in a humidified incubator (5% carbon dioxide and 95% air). The platelets were diluted approximately 300-fold to achieve the desired platelet-to-virus ratio for the assay. Platelet washing was not included to avoid additional manipulation (repeated centrifugation and resuspension) prior to viral stimulation. We used RPMI and 2% heat inactivated FCS, as recommended by EVAg [SARS-CoV-2 virus, strain hCoV-19/Italy/LAZ-INMI1-isl/2020 (clade O, lineage B) (17, 18)] during the short infection/adsorption period to minimize serum mediated inhibition of viral adsorption/entry, as lowering serum to 2% reduces serum proteins that can bind or partially neutralize virions (19, 20). FCS was used instead of human serum that would contain several of same proteins as also released from *in vitro* stimulated platelets. After incubation, the samples were centrifuged at 13000 g for 5 min. Platelet-free plasma was transferred to Eppendorf tubes and stored at −30°C until protein measurements by the same EIAs as described above. In all experiments we included an unstimulated control and the Protease-Activated Receptor (PAR) 1 agonist, SFLLRN (f. c. 100 µM, synthesized at The Biotechnology Centre of Oslo, Norway) as a positive control for platelet activation.

### Statistical methods

Continuous normally distributed demographic variables were compared with Student's t-test and presented as mean ± SD, whereas non-normally distributed variables were presented as median (25th/75th percentile) and compared with the Mann–Whitney *U* test. Categorical data were compared using the chi-square test.

For comparing groups during 10-day hospitalization, groups were compared at each timepoint with a general linear model with platelet-derived biomarkers as dependent, group (i.e., moderate disease vs. ICU/RF; Survivors vs. non-Survivors; men vs. women; users of anti-coagulants and dexamethasone vs. non-users) as fixed, and age, sex, dexamethasone, anticoagulant use, comorbidities, platelet count as covariates. When assessing effects on platelet counts, these counts were omitted from the models and similarly, dexamethasone and anti-coagulant use were omitted as covariates from their respective models when used as fixed factors. Data are presented as estimated marginal means and 95% confidence intervals.

The association between admission levels of platelet-derived mediators and 60-day all-cause mortality was first assessed by receiver-operating characteristic (ROC) analysis. Significant markers were dichotomized (median) and association with outcome was further assessed by the Kaplan–Meier analysis. For analysis of 60-day mortality in an adjusted Cox regression model, normalized log-values (log/standard deviation) were used, and adjustment covariates were included one-by-one (age, P/F ratio, comorbidities, CRP, platelet counts, and use of anti-coagulants).

For comparing levels of platelet-derived biomarkers in COVID-19 patients at three, six and 12-month follow-up, a multivariate general linear model was used with group (healthy control or COVID patients) as fixed and age, sex and comorbidity (information about anticoagulation and platelet count was not available from the follow-up period) as covariates.

*The vitro* experiments examining the effect of inactivated SARS-CoV-2 on platelet release, data were analyzed by One-way ANOVA. Dunnett's multiple comparison test was used to compare difference with unstimulated platelets.

Simple correlations were assessed by Spearman. Statistics were performed using SPSS version 29.0.0.0. A two-sided *p*-value of <0.05 was deemed statistically significant.

## Results

### Baseline characteristics of the study cohort

As shown in Table 1, patients in the most severe outcome group (ICU admitted and/or RF) were older than the other patients, had higher BMI, and were more often obese. The ICU/RF patients were in greater need of oxygen therapy and dexamethasone treatment, and had significantly higher levels of ferritin, CRP and WBC than the other patients.

**Table 1:** Demographic and clinical baseline values in healthy controls and patients admitted to hospital with SARS-CoV-2 infection.

Parameter	Healthy *n* = 29	COVID-19 *n* = 245	*P*	Moderate disease *n* = 146	ICU/RF *n* = 99	*P*
Age, years	63.1 ± 7.3	57.2 ± 15.2	0.021	55.1 (16.4)	60.3 (12.8)	0.004
Male sex, no (%)	13 (44.8)	147 (60)	0.12	85 (58.2)	62 (62.6)	0.49
BMI, kg/m2	24.7 ± 3.0	28.8 ± 4.9	<0.001	28.2 ± 4.8	29.6 ± 4.8	0.022
Obesity, no (%)	2 (6.9)	86 (35.1)	0.002	42 (28.8)	44 (44.4)	0.012
Symptom duration, days	0	8.4 ± 5.6	NA	8.5 ± 5.8	8.3 ± 5.3	0.38
Oxygen therapy, days	0	7 (3–12)	NA	3 (1–5)	12 (8–20)	<0.001
Dexamethasone, no (%)	0	138 (56.3)	NA	57 (39)	81 (81.8)	<0.001
Anticoagulants, no (%)	0	215 (87.8)	NA	119 (81.5)	96 (97.0)	<0.001
Cardiovascular disease, no (%)	0	42 (17.1)	0.16	23 (15.8)	19 (19.2)	0.48
Hypertension, no (%)	0	85 (34.8)	<0.001	47 (32.4)	38 (38.4)	0.34
Chronic pulmonary disease, no (%)	0	21 (8.6)	0.10	11 (7.5)	10 (10.1)	0.48
Asthma, no (%)	0	46 (18.8)	0.011	28 (19.2)	18 (18.2)	0.85
Renal, no (%)	0	20 (8.2)	0.11	10 (6.8)	10 (10.1)	0.36
Chronic neurological disease, no (%)	0	12 (4.9)	0.22	8 (5.5)	4 (4.0)	0.61
Cancer, no (%)	0	11 (4.5)	0.24	6 (4.1)	5 (5.1)	0.73
Diabetes, no (%)	0	58 (23.7)	0.003	35 (24.0)	23 (23.2)	0.89
Comorbidities^†^	0	185 (75.5)	<0.001	105 (71.9)	80 (80.8)	0.50
Death 60	0	26 (10.6)	NA	3 (2.1)	23 (23.2)	<0.001
Hemoglobin, g/dL	13.8 ± 1.1	12.9 ± 1.7	0.003	13.1 ± 1.7	12.7 ± 1.7	0.055
WBC, *10^9^ /L	5.6 ± 1.3	6.8 ± 3.5	0.025	6.1 ± 2.8	7.9 ± 4.0	<0.001
Lymphocytes*10^9^ /L	1.7 ± 0.4	1.1 ± 0.6	<0.001	1.2 ± 0.6	0.8 ± 0.4	<0.001
Neutrophils*10^9^ /L	3.2 ± 0.9	5.3 ± 3.4	<0.001	4.4 ± 2.7	6.7 ± 3.8	<0.001
Platelets, *10^9^ /L	284.5 ± 66.0	224.5 ± 87.6	<0.001	230.1 ± 87.0	216.3 ± 88.3	0.12
Creatinine, µmol/L	69 (65, 82)	72 (58, 86)	0.86	72 (59, 84)	70 (58, 87)	0.90
CRP mg/L	1.0 (0.8, 1.4)	55 (24, 120)	<0.001	43 (15, 102)	89 (46, 152)	<0.001
Ferritin µg/L	–	600 (291, 1,062)	–	477 (209, 833)	852 (455, 1,479)	<0.001

Continuous data are given as mean ± SD or median (25th, 75th) percentile. BMI, body mass index; CRP, c-reactive protein; ICU, intensive care unit; WBC, white blood cells. Comorbidities^†^ represents accumulated comorbidities + obesity. None of the controls had any clinical symptoms of ongoing disease and none was using any medication including dexamethasone and anticoagulants. In total, 215 patients were treated with anticoagulants (all but six used heparin). NA, not assessed.

### Levels of platelet-derived mediators in relation to ICU admission and/or the development of respiratory failure

**Figure 1:**
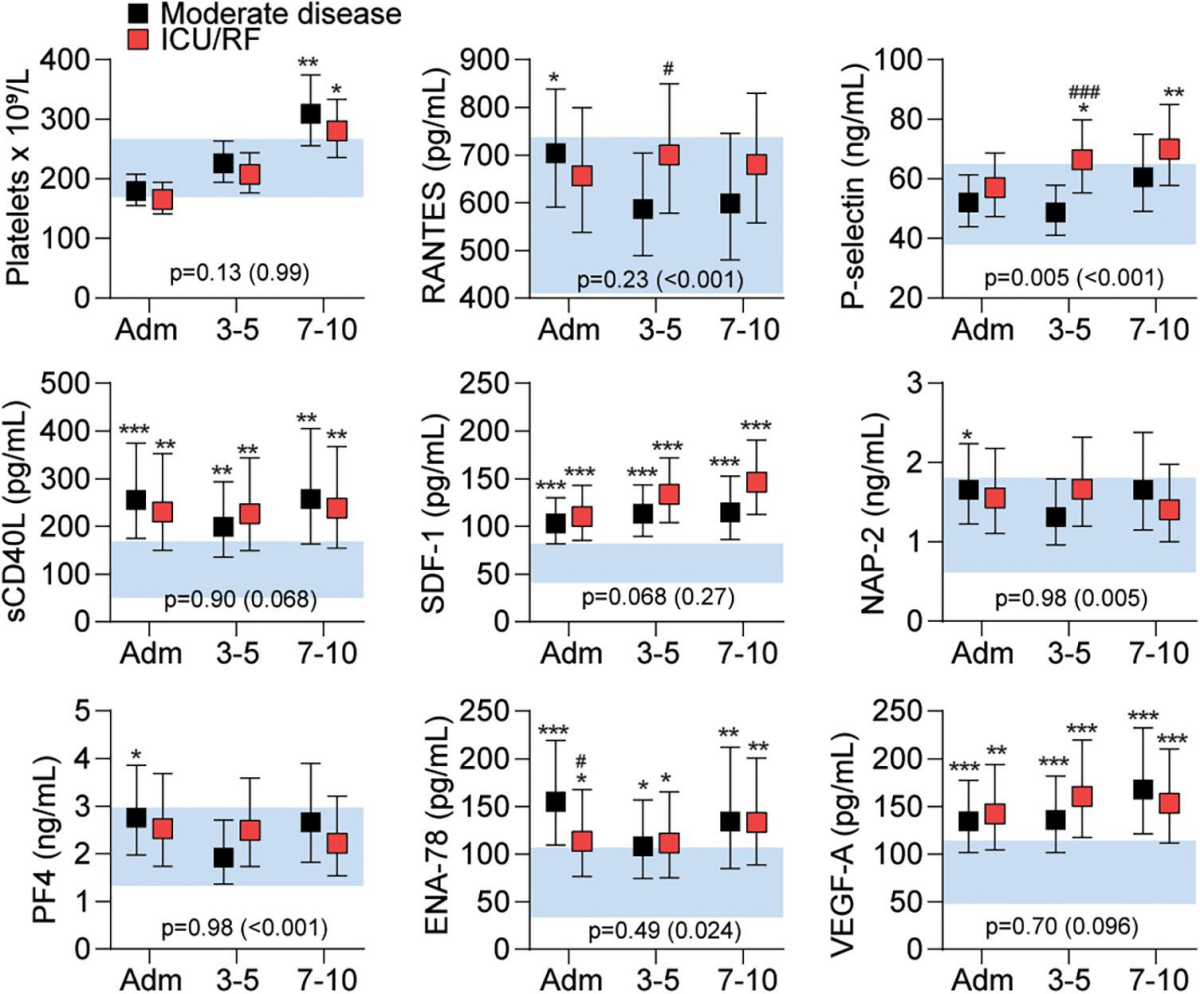
Platelet counts and platelet-derived mediators in patients during the acute phase of COVID-19. Data are shown as estimated marginal means and 95% confidence intervals (CI). The blue shaded area shows the 95% CI for platelet-derived mediators in healthy controls (*n* = 29). The *p*-values reflect the group (moderate disease or ICU/RF), and group × time (in brackets) from linear mixed models adjusted for age, sex, dexamethasone, anticoagulation, platelet count and comorbidity. *p < 0.05, **p < 0.01, ***p < 0.001 vs. HC, ^#^*p* < 0.05, ^###^*p* < 0.001 ICU/RF vs. moderate disease. ENA-78, epithelial neutrophil-activating protein 78; NAP-2, neutrophil-activating peptide-2; PF4, platelet factor 4; RANTES, *R*egulated on *A*ctivation, *N*ormal *T*-cell *E*xpressed and *S*ecreted; SDF-1, stromal cell-derived factor 1; VEGF-A, vascular endothelial growth factor A.

Figure 1 shows the temporal plasma profile of platelet-derived mediators and platelet counts in relation to the combined endpoint of ICU admission and/or RF after adjustment for age, sex, comorbidities, dexamethasone treatment, platelet counts (omitted when calculating platelet counts), and anti-coagulation use.

During hospitalization, a gradual increase in platelet counts were observed, reaching levels above healthy controls at 10 days following hospital admission, but with no relation to disease severity as assed by development of RF or the need of treatment at ICU (Figure 1). For the platelet-derived mediators, we found a more diverse pattern. First, plasma levels of sCD40L, SDF-1, and VEGF-A were persistently elevated during hospitalization compared with healthy controls, but we found no relation to disease severity. Second, levels of PF4 and NAP-2 were elevated compared with healthy controls only at hospital admission, reaching statistical significance in the less severely diseased patients, but with no significant differences between the two severity groups at any time-point during hospitalization. Finally, in contrast to the other markers, RANTES, P-selectin, and ENA-78 showed some relation to disease severity. Whereas plasma levels of RANTES and P-selectin were significantly *higher* in the ICU/RF group as compared with the less severe patient group at 3–5 days following hospital admission, plasma levels of ENA-78 were significantly *lower* in the patients with the most severe disease at hospital admission.

### Platelet-derived mediators in relation to 60-day mortality

**Figure 2:**
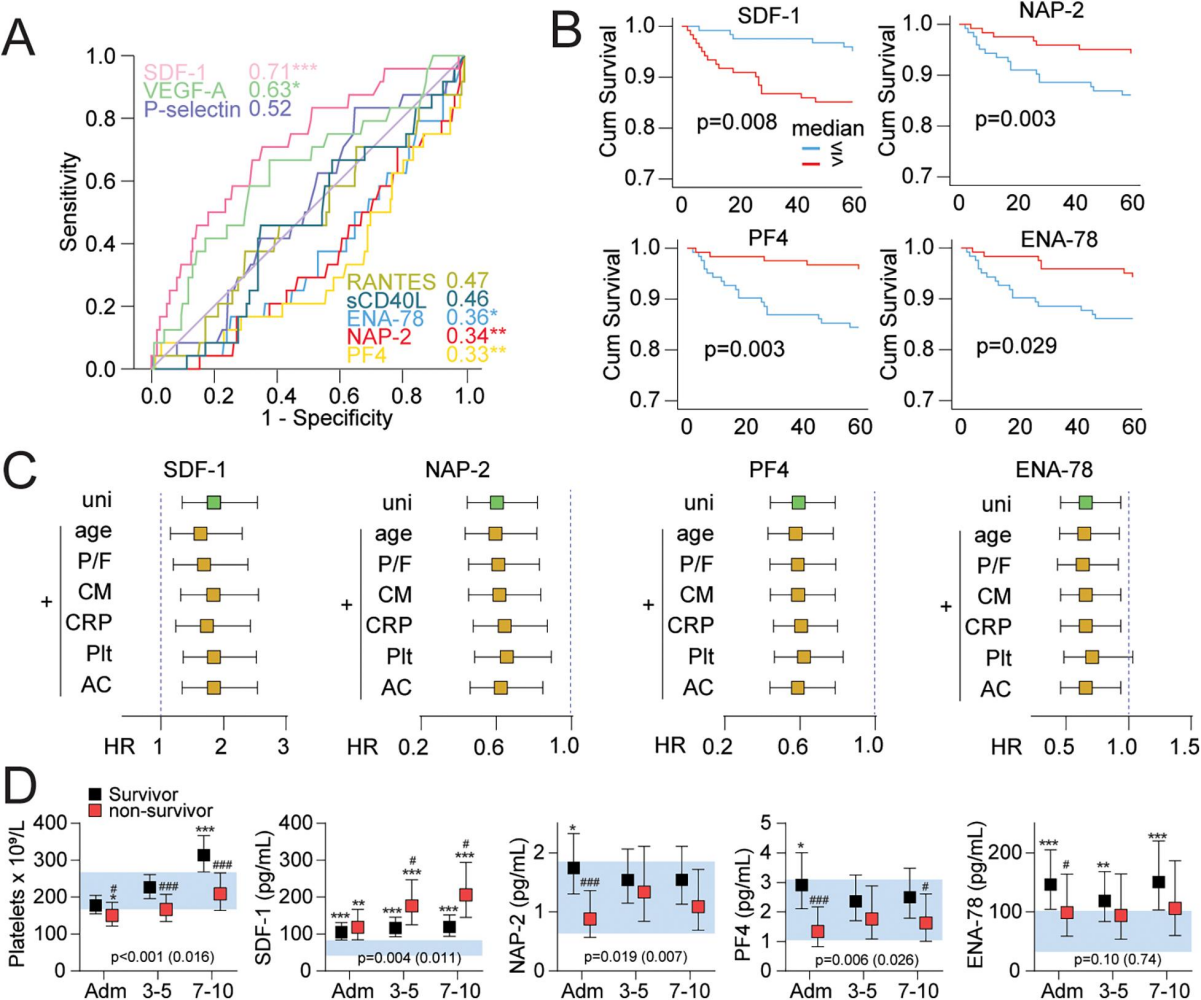
Increased mortality with increased SDF-1 and decreased ENA-78, NAP-2 and PF4. **(A)** Receiver operating characteristics (ROC) analysis and area under curves (AUCs) for the markers. *p < 0.05, **p < 0.01, ***p < 0.001. **(B)** Kaplan–Meier analysis of mortality at 60 days based on dichotomized median values of markers found significant in the ROC analysis. The cumulative survival between VEGF-A high/low groups was not significantly different in Kaplan–Meier analyses (not shown). **(C)** Cox regression of admission levels of platelet-related biomarkers based on normalized log-values (log/standard deviation). Data are shown as hazard ratios (HR) and 95% confidence intervals (CI) for each marker as univariate (uni) and with one-by-one inclusion of covariates. P/F, partial pressure of oxygen in arterial blood (PaO2) divided by the fraction of inspired oxygen (FiO2). **(D)** Temporal profile of platelet-related biomarkers during the first 10 days of hospitalization according to 60-day mortality shown as estimated marginal means and 95% confidence intervals (CI). The blue shaded area shows the 95% CI for platelet-related biomarkers in healthy controls. The *p*-values reflect the group (survivor vs. non-survivor), and group × time (in brackets) from linear mixed models adjusted age, sex, dexamethasone, anticoagulation, platelet count and comorbidity. *p < 0.05, **p < 0.01, ***p < 0.001 vs. HC, ^#^*p* < 0.05, survivor vs. non-survivor. AC, anticoagulant use; CM, accumulated comorbidities including obesity; CRP, C-reactive protein; ENA-78, epithelial neutrophil-activating protein 78; NAP-2, neutrophil-activating peptide-2; PF4, platelet factor 4; Plt, platelet count; RANTES, *R*egulated on *A*ctivation, *N*ormal *T*-cell *E*xpressed and *S*ecreted; SDF-1, stromal cell-derived factor 1; VEGF-A, vascular endothelial growth factor A.

During follow-up, 26 of the 245 patients died within 60 days after hospital admission. The non-survivors were older, had a higher proportion of men, at admission they had lower BMI, were more likely to receive oxygen and dexamethasone therapy, had higher levels of CRP and ferritin, lower hemoglobin levels, lower lymphocyte and platelet counts, and had more co-morbidities, in particular cardiovascular disorders and chronic pulmonary diseases (Supplementary Table S1). ROC analyses showed that *high* admission levels of SDF-1 and *low* admission levels of PF4, ENA-78 and NAP-2 were associated with increased mortality (Figure 2A), as also demonstrated by the Kaplan–Meier analyses (Figure 2B). Admission levels of the other platelet-derived mediators were not significant associated with mortality. Cox regression analyses confirmed this pattern with *high* admission levels of SDF-1 and *low* admission levels of PF4, ENA-78 and NAP-2 associated with 60-day all-cause mortality (Figure 2C). Importantly, these associations were only to a lesser degree influenced by adjusting for relevant co-variants such as CRP, the use of anticoagulants, platelet counts, the degree of respiratory failure (i.e., P/F ratio), and co-morbidity (Figure 2C). Figure 2D shows the association between the temporal profile of levels of platelet count, SDF-1, PF4, ENA-78 and NAP-2 during hospitalization in relation to 60-day mortality. Patients who died had significantly lower platelet counts at all time-points during hospitalization as compared to those who survived. Further, whereas the association of *low* levels of PF4, ENA-78 and NAP-2 and mortality seemed to be restricted to baseline levels (except for a significant, but weaker association of low levels of PF4 at seven to ten days after hospital admission), the association of *high* levels of SDF-1 with mortality was also observed at three to five and seven to ten days after hospitalization (Figure 2D).

### Correlation of platelet-derived mediators and platelet counts, CRP, and D-dimer

**Figure 3:**
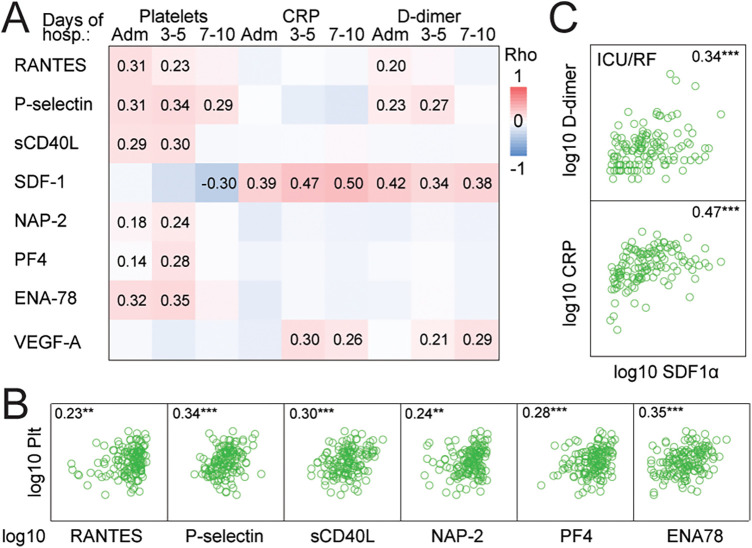
Association between platelet-derived mediators and platelet counts, CRP and D-dimer. **(A)** Correlation between platelet-derived mediators at different timepoints (admission, 3–5 days, and 7–10 days, respectively) and platelet count, CRP and D-dimer, respectively. Correlations were calculated with the Spearman correlation coefficient (rho) and only significant correlations are displayed with coefficients. Positive correlation indicated by red, negative with blue. **(B)** Scatterplots showing correlation between platelet counts and selected platelet-derived mediators at 3-5 days. **(C)** Scatterplots showing correlation between SDF-1 and CRP and D-dimer at 3-5 days. Spearman's rho is given in the graph for each correlation. **p < 0.01, ***p < 0.001. Adm, admission; CRP, C-reactive protein; ENA-78, epithelial neutrophil-activating protein 78; NAP-2, neutrophil-activating peptide-2; PF4, platelet factor 4; RANTES, *R*egulated on *A*ctivation, *N*ormal *T*-cell *E*xpressed and *S*ecreted; SDF-1, stromal cell-derived factor 1; VEGF-A, vascular endothelial growth factor A.

Figure 3A shows the correlations of the platelet-derived mediators with platelet counts, CRP, and D-dimer during hospitalization. Positive correlations with platelet counts were observed for RANTES, P-selectin, sCD40L, NAP-2, PF4 and ENA-78. These correlations were strongest at three to five days (shown as scatterplots in Figure 3B) and did not persist at 7–10 days except for P-selectin. As for CRP and D-dimer, these consistently correlated positively with SDF-1 (shown as scatterplot in Figure 3C at 3–5 days), and to a lesser degree with VEGF-A. Finally, D-dimer correlated positively with RANTES and P-selectin at early time-points.

### The effects of anticoagulant and dexamethasone on platelet-derived mediators

Out of the 245 patients 215 were treated with anticoagulants (209 used heparin, 5 used Direct Oral Anticoagulant and 1 used warfarin) and 138 (56.3%) received dexamethasone in line with standard management of severely diseased COVID-19 patients from autumn 2020 (21). Although adjustment for these medications did not influence our mortality analyses, we examined whether they influenced the levels of the actual platelet-derived mediators during hospitalization. As illustrated in Supplementary Table S2, only minor associations with these treatments were observed for the platelet-derived mediators. Higher P-selectin and SDF-1 at 3–5 days were observed in those who received dexamethasone while no effects were seen for anti-coagulant use on markers levels. However, a significant increase in platelet count was observed in anti-coagulant users at 3–5 and 7–10 days. Caution is needed when interpreting this effect as number of observations towards the end of the acute phase were much lower (i.e., at 7–10 days, 88 used heparin and 8 did not use).

### Platelet-derived mediators after discharge

In a subpopulation of the 245 patients, we also analyzed plasma levels of the platelet-derived mediators at three, six and 12 months after hospitalization. Demographic and clinical admission values in patients with (*n* = 176) and without (*n* = 114) samples at follow-up are shown in Supplementary Table S3. Significant differences were lower age, shorter symptom duration, more comorbid renal and chronic neurological disease, lower hemoglobin and lower lymphocyte counts in patients with follow-up samples.

**Figure 4:**
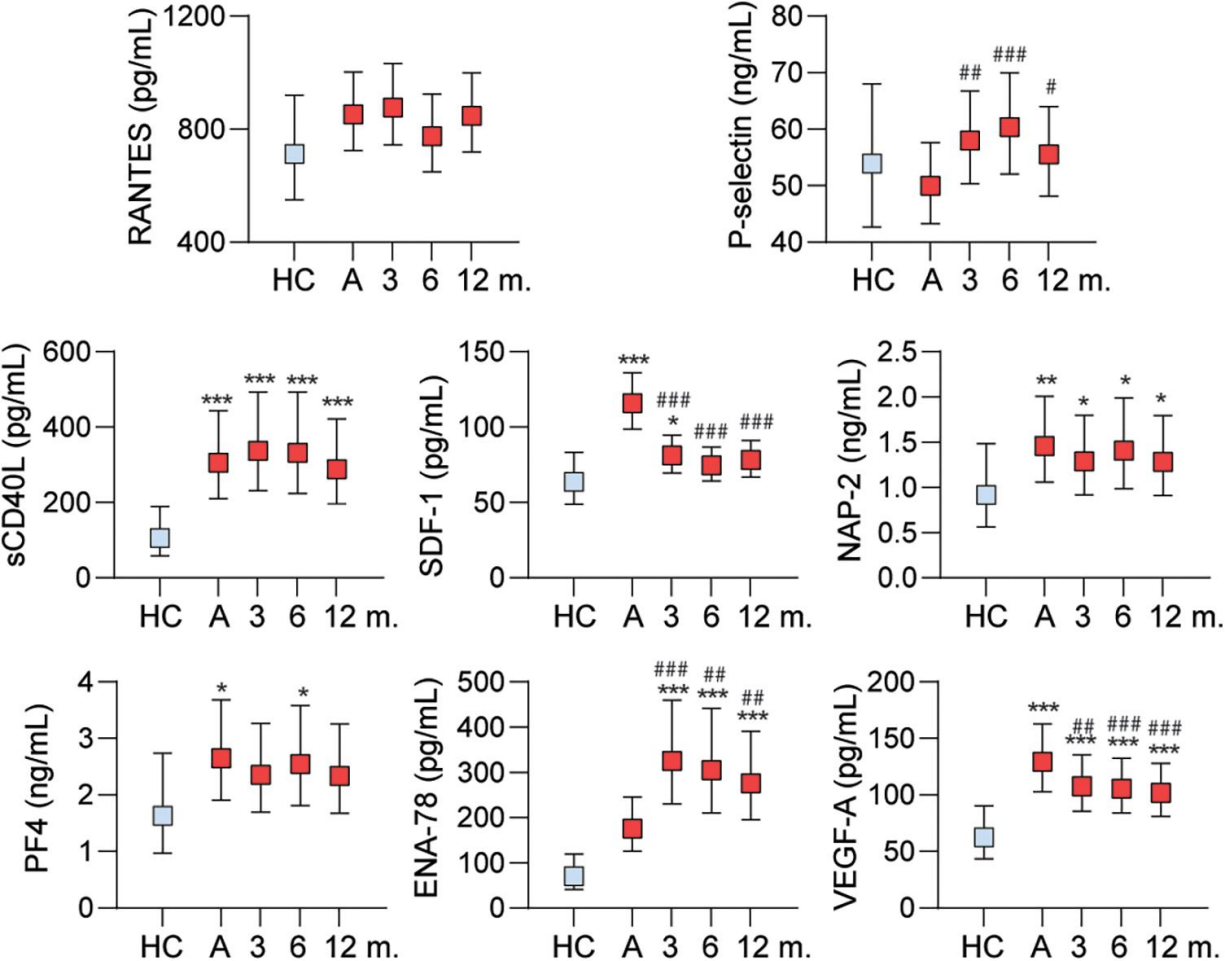
Follow-up data of the platelet-related mediators at admission (three (*n* = 151), six (*n* = 68) and 12 (*n* = 67) months. Analyzed by multiple general linear models with covariates age, sex, and comorbidity (information about anticoagulation and platelet count was not available from the follow-up period). *p < 0.05, **p < 0.01, ***p < 0.001 vs. healthy controls (HC). #*p* < 0.05, ##*p* < 0.01, ###*p* < 0.001 vs. admission level. ENA-78, epithelial neutrophil-activating protein 78; NAP-2, neutrophil-activating peptide-2; PF4, platelet factor 4; RANTES, *R*egulated on *A*ctivation, *N*ormal *T*-cell *E*xpressed and *S*ecreted; SDF-1, stromal cell-derived factor 1; VEGF-A, vascular endothelial growth factor A.

Whereas most of the mediators were within levels in healthy controls, sCD40L, ENA-78 and VEGF-A were markedly elevated in COVID-19 patients at all-time points (Figure 4). More modest elevations were also observed for NAP-2, PF4 and SDF-1 at some time-points.

As for changes from admission levels in patients with COVID-19, an increase was seen during follow-up for P-selectin and ENA-78, while SDF-1 and VEGF-A decreased (Figure 4).

### Platelet-derived mediators in males and females

It is well known that immune responses can be sex dependent. Thus, although our results were adjusted for sex differences, we examined whether the major findings showed differences between males and females. As shown in Supplementary Table S4, some significant findings were revealed. First, whereas males had higher levels of SDF-1 during hospitalization, females had higher levels of ENA-78. Finally, ENA-78 was higher in women at three months.

### The effects of inactivated SARS-CoV-2 on the release of platelet-derived mediators *in vitro*

**Figure 5:**
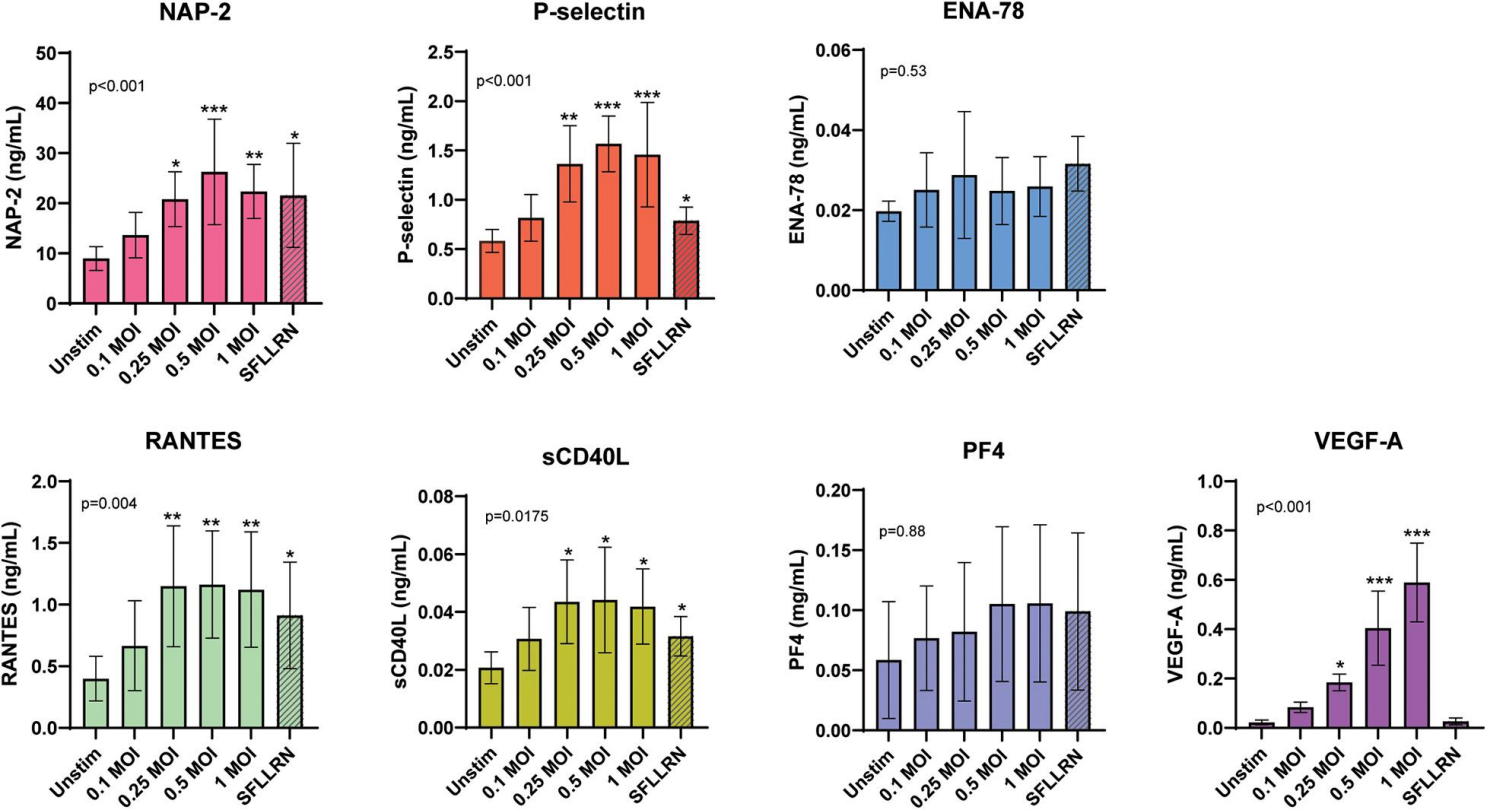
*In vitro* stimulation of platelets from healthy controls (*n* = 6) with increasing doses of inactivated SARS-CoV-2. Analyzed by one-way ANOVA and data shown as means ± SD. *p < 0.05, **p < 0.01, ***p < 0.001 vs. unstimulated control. In all experiments we included an unstimulated control and the Protease-Activated Receptor (PAR) 1 agonist, SFLLRN (f. c. 100 µM) as a positive control for platelet activation. MOI: multiplicity of infection. ENA-78, epithelial neutrophil-activating protein 78; NAP-2, neutrophil-activating peptide-2; PF4, platelet factor 4; RANTES, *R*egulated on *A*ctivation, *N*ormal *T*-cell *E*xpressed and *S*ecreted; VEGF-A, vascular endothelial growth factor A.

To clarify whether the altered levels of the platelet-derived mediators also could be a result of a direct effect of SARS-CoV-2 on platelets, we measured the release of the mediators after stimulating platelets with different concentrations of inactivated SARS-CoV-2. In a pilot experiment we performed both dose- and time-response assessments. Although some differences (Supplementary Figure S1), we decided to use 4 h as the incubation time in the main experiment. For comparison, we also included the PAR-1 agonist SFLLRN as a positive control and unstimulated platelets as a negative control in each experiment. Figure 5 shows that SARS-CoV-2 dose-dependently induced release of NAP-2, P-selectin, RANTES, sCD40L and VEGF-A. In contrast, there was no significant increase in the release of ENA-78 and PF4. As for SDF-1, we did not detect any release after SARS-CoV-2 nor for SFLLRN exposure (data not shown).

## Discussion

In the present study, we examined the regulation of platelet-derived mediators during hospitalization for COVID-19 emphasizing the relation to clinical outcomes and the levels of these molecules several months post-hospitalization. Our main findings were: (i) Whereas *high* levels of SDF-1 were associated with adverse outcomes defined as the need for treatment at ICU and/or RF and 60-day total mortality, *low* levels of PF4, ENA-78 and NAP-2 were associated with 60-day total mortality and for ENA-78, *low* levels were also associated with ICU/RF. (ii) Although most of the mediators were normalized after hospitalization, plasma levels of sCD40L, ENA-78, and VEGF-A were markedly elevated compared to healthy controls for up to 12 months after hospitalization, and for SDF-1 at three months. (iii) Whereas women in general had elevated levels ENA-78 compared with males, SDF-1 levels were higher in men. (iv) *In vitro*, inactivated SARS-CoV-2 induced the release of several of the examined mediators, while SDF-1 was not detectable under any of the conditions. Our findings underscore the role of platelet-derived mediators in the pathogenesis of COVID-19, potentially involving direct effects of SARS-CoV-2.

Of the examined molecules, only PF4 is platelet specific. However, all the other markers are, although in a different degree, platelet-derived. Moreover, the cellular expression of the examined molecules may not necessarily be reflected in their plasma concentration where platelets may be an important contributor even if other cells express the molecules at a higher level. Nonetheless, the other measured molecules (than PF4) differ in the contribution of platelets as a source of plasma levels. Thus, for NAP-2 and P-selectin, platelets seem to be the major cellular source (22, 23). Moreover, even if CD40L and RANTES are highly expressed in T cells, platelets seem to be the major contributor to plasma levels of soluble CD40L and potentially also RANTES (22, 24). Whereas ENA-78 is strongly expressed in epithelial cells and endothelial cells, platelets also contribute to its plasma levels (25). Finally, plasma levels VEGF-A and in particular SDF-1 have several additional cellular sources (26, 27). Indeed, the different pattern of SDF-1 as compared with PF4, ENA-78 and NAP-2 in relation to mortality, i.e., high SDF-1 levels and low levels of PF4, ENA-78 and NAP-2 are associated with mortality, most probably reflect different cellular sources of plasma levels; multiple cells vs. platelet as the major source, respectively.

Some studies have shown that low platelet counts are associated with adverse outcomes in hospitalized COVID-19 disease (9–11). In COVID-19, plasma levels of P-selectin has been associated with disease severity and thromboembolic events (28, 29), sCD40L has been suggested as an early marker of severe disease (30), meta-analyses suggest a prognostic potency for VEGF (31), and for RANTES, an association with mild vs. severe disease have been reported (32). However, data on other platelet-derived mediators are scarce or lacking. In the present study, we extend previous studies by showing that low levels of PF4, ENA-78 and NAP-2 are associated with 60-day total mortality in hospitalized COVID-19 patients. This pattern with low levels associated with poor prognosis may potentially reflect that platelets over time have been activated, resulting in platelet exhaustion as also seen during other conditions with severe inflammation such as during septicemia (33). A support of this interpretation is that low and not high levels of PF4, ENA-78 and NAP-2 were associated with increased mortality, potentially indicating persistently activated and exhausted platelets. A similar pattern with a reduced platelet granule release capacity has also been showed by others in severely diseased COVID-19 patients (34, 35). Moreover, the most activated platelets will interact with endothelial cells and even infiltrate adjacent inflamed tissue (36), further contributing to low plasma levels of platelet-derived mediators during severe inflammation.

Importantly, these platelet-derived mediators may not only contribute to endothelial dysfunction and thromboembolism but may more directly be involved in pulmonary inflammation and damage. Thus, PF4, being a potent chemoattractant for neutrophils, has been shown to promote pulmonary tissue inflammation in influenza (37). Also, ENA-78 has been reported to promote lung inflammatory innate immune response in a mouse model infected with SARS-CoV-2 (38). Moreover, NAP-2 seems to be directly involved in severe pulmonary conditions like ARDS (39). Thus, the platelet-derived mediators that were associated with adverse outcomes could not only be related to adverse thromboembolic events, but also more directly contribute to pulmonary pathology in COVID-19.

The activation of platelets during COVID-19 could be secondary to systemic inflammation, and the increase in platelet counts in those who received dexamethasone may support such a notion. In addition, however, several studies have since the start of the pandemic suggested that SARS-CoV-2 may directly activate platelets. However, the disparate observations of viral load within platelets from COVID-19 patients, coupled with the identification of numerous alternative receptors on the platelet surface, have contributed to some uncertainty (6). Herein, we show that inactivated SARS-CoV-2 *in vitro* increased the release of several of the examined mediators. These findings may potentially support a contributing role of SARS-CoV-2-platelet interaction in the persistent inflammation characterizing severe COVID-19.

In contrast to the pattern for PF4, ENA-78 and NAP-2, high levels of SDF-1 were associated with adverse outcomes in hospitalized COVID-19 patients. The reasons for this pattern may have several explanations. In addition to the differences in cellular sources as discussed above, we used an EIA that measured the two main isoforms of SDF-1, *α* and *β*, both interacting with CXCR4. However, the *β* isoform is more resistant to proteolysis in circulation and will accordingly circulate in higher levels, and is primarily released from highly vascularized tissue and its release from platelets is somewhat unclear (40). The latter could potentially have contributed to the lack of SARS-CoV-2 induction of SDF-1 release from platelets in our *in vitro* experiments. Additionally, SDF-1 released from platelets has an autocrine effect as it remains bound to the platelet surface through CXCR4 on the platelet surface (41). Nonetheless, SDF-1 binding to CXCR4 has been involved in the development of pulmonary fibrosis (42), pulmonary hypertension (43) and pulmonary inflammation in ARDS (44). Moreover, high levels of SDF-1 have been related to disease severity (45) and therapy targeting SDF-1 in severe COVID-19 has been suggested (46). Very recently, however, it was suggested that SDF-1 ameliorates neutrophilia and disease severity in SARS-CoV-2 infection (47), but to prove this intriguing hypothesis, that the increased SDF-1 levels represent a counteracting mechanism, will have to be confirmed in further studies.

Somewhat surprisingly, several months after hospital admission i.e., three, six and 12 months, hospitalized COVID-19 patients had persistently elevated levels of sCD40L, ENA-78 and VEGF-A, and to some degree also of NAP-2, PF4 and SDF-1, compared to healthy controls. We and others have underscored the potential risk for cardiovascular events following COVID-19 (48, 49), and notably, several of these markers have been related to cardiovascular disease (50–53). However, although we found elevated levels of some of the measured markers for up to 12 months after hospital admission as compared with healthy controls, this could potentially be related to increased proportion of co-morbidities in the patient group as compared with healthy controls. Indeed, recent studies suggest that comorbidities like cardiovascular diseases including hypertension as well as metabolic diseases will increase the risk and severity of long COVID symptoms (54, 55).

Interestingly, whereas males had higher levels of SDF-1 during hospitalization and the relation of this mediator to adverse outcome was mostly observed in males, females had in general higher levels of PF4, ENA-78 and NAP-2 than males after hospitalization. This pattern could suggest that females are more prone to platelet activation and release of platelet-related mediators than males, as also suggested in previous non-COVID studies (56). If this is confirmed in larger studies, it could have consequences for sex-specific preventive options following COVID-19

The present study has some limitations such as moderate number of patients, lack of data from infected organs, and use of inactivated rather than live virus. Moreover, we lack data on the more recent SARS-CoV-2 strains such as the omicron variants. Also, although our procedures aimed to eliminate non-viral components in the *in vitro* experiment, we cannot exclude that such proteins could have contributed to the release of the examined molecules from SARS-CoV-2-exposed platelets. In addition, we lack data on platelet viability and responsiveness as assessed by flow cytometry at the end of the *in vitro* experiment. Furthermore, we do not have any data on long COVID symptomatology or other forms of long-term outcomes which limit our findings on platelet-derived markers after hospitalization. Finally, while PF4 is platelet specific and all the other markers are, although in a different degree, platelet-derived, we cannot exclude that other cell types have contributed to their plasma levels, in particular to levels of SDF-1 and in some degree also levels of VEGF-A where other cellular sources are of more importance (26, 27). However, the study has a well-characterized cohort, measuring a broad spectrum of platelet-derived mediators in relation to well-defined clinical outcomes and the combination of *in vivo* and *in vitro* studies is also a strength of the study.

Our findings underscore a pathogenic role of platelet activation with release of several inflammatory mediators in severe COVID-19, showing a different pattern of “classical” platelet-derived mediators as opposed to SDF-1. The study also points to a persistent platelet activation following hospitalization. These findings could imply the need for therapy to prevent cardiovascular disease following COVID-19.

## Data Availability

The raw data supporting the conclusions of this article will be made available by the authors, without undue reservation.

## Supplementary material

Supplementary Figure S1 *In vitro* stimulation [for 1 (blue) or 3 (red) hours, respectively] of platelets from healthy controls (*n* = 2) with increasing doses of inactivated SARS-CoV-2. In all experiments we included an unstimulated control and the Protease-Activated Receptor (PAR) 1 agonist, SFLLRN (f. c. 100 µM) as a positive control for platelet activation. MOI: multiplicity of infection. ENA-78, epithelial neutrophil-activating protein 78; PF4, platelet factor 4; RANTES, *R*egulated on *A*ctivation, *N*ormal *T*-cell *E*xpressed and *S*ecreted.
