# Supplementary figures and images for: Platelet-derived mediators in hospitalized COVID-19 patients and associations to respiratory failure, ICU admittance and 60-day mortality
Source: Front Cardiovasc Med. 2026 Feb 25;13:1685861. doi: 10.3389/fcvm.2026.1685861 (PMC12976018; doi:10.3389/fcvm.2026.1685861)

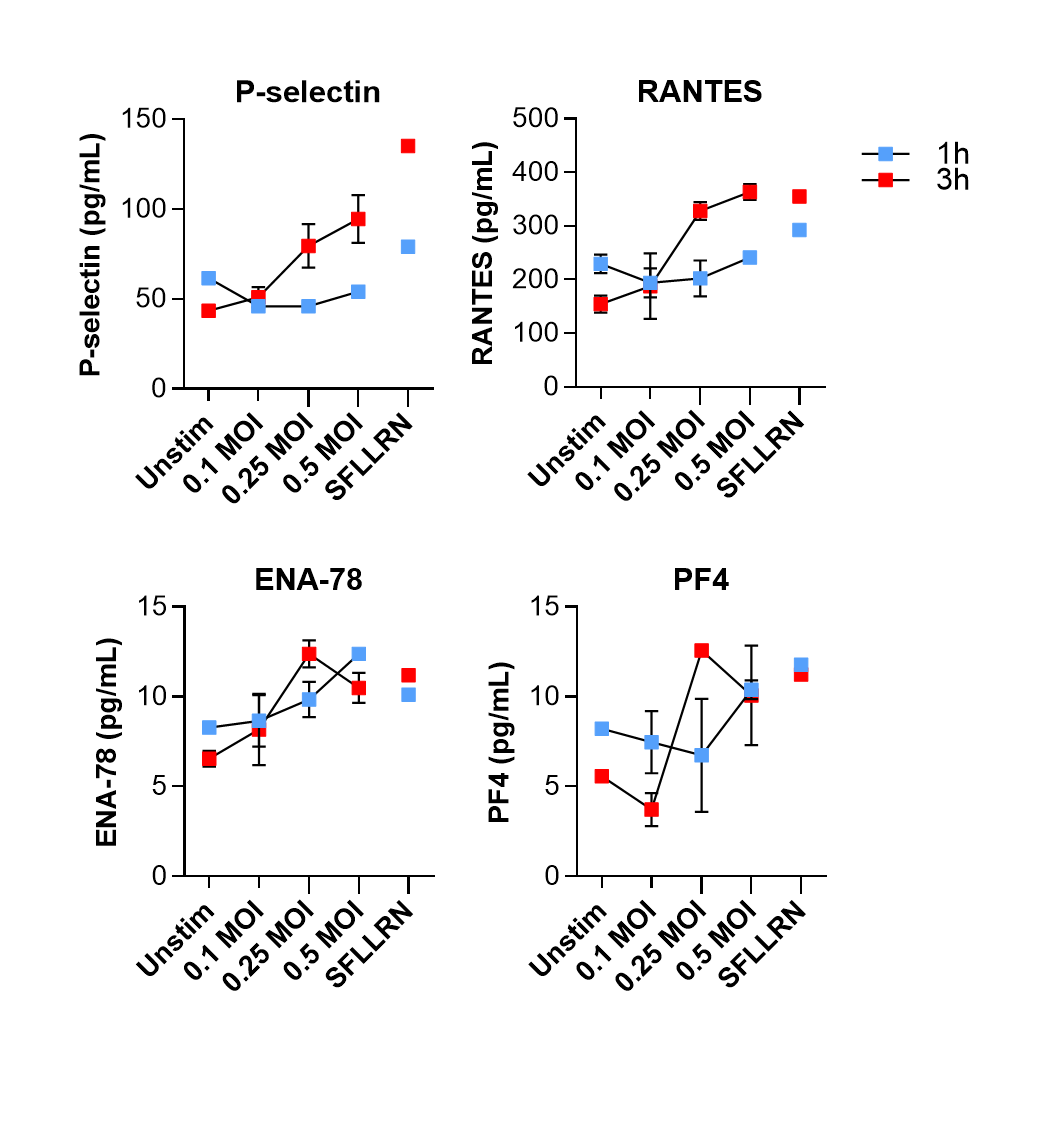

Supplement: Supplementary Figure S1 — In vitro stimulation [for 1 (blue) or 3 (red) hours, respectively] of platelets from healthy controls (n = 2) with increasing doses of inactivated SARS-CoV-2. In all experiments we included an unstimulated control and the Protease-Activated Receptor (PAR) 1 agonist, SFLLRN (f. c. 100 µM) as a positive control for platelet activation. MOI: multiplicity of infection. ENA-78, epithelial neutrophil-activating protein 78; PF4, platelet factor 4; RANTES, Regulated on Activation, Normal T-cell Expressed and Secreted. [file Image1.tif]
