# Supplementary material for: Platelet-derived mediators in hospitalized COVID-19 patients and associations to respiratory failure, ICU admittance and 60-day mortality
Source: Front Cardiovasc Med. 2026 Feb 25;13:1685861. doi: 10.3389/fcvm.2026.1685861 (PMC12976018; doi:10.3389/fcvm.2026.1685861)
Supplement: Supplementary file 2 [file Table1.docx]

Supplementary Table 1

Demographic and clinical admission values in patients admitted to hospital with SARS-CoV-2 infection who either survived or died within 60 days.

|  | Survivors  n = 219 | Non-survivors  n = 26 | p |
| --- | --- | --- | --- |
| Age, years | 55.9±15.1 | 68.2±11.4 | <0.001 |
| Male sex, no (%) | 126(57,5) | 21(80.8) | 0.022 |
| BMI, kg/m2 | 29.9±5.0 | 26.8±3.7 | 0.028 |
| Obesity, no (%) | 81(37.0) | 5(19.2) | 0.073 |
| Symptom duration, days | 8.6±5,7 | 6.8±3.9 | 0.075 |
| Oxygen therapy, days | 5.5(3-10.5) | 14(10-27) | <0.001 |
| Dexamethasone, no (%) | 117(53.4) | 21(80.8) | 0.008 |
| Anticoagulants total, no (%) | 192 (87.7) | 23 (88.5) | 0.91 |
| Cardiovascular disease, no (%) | 31(14.2) | 11(42.3) | <0.001 |
| Hypertension, no (%) | 77(35.3) | 8(30.8) | 0.65 |
| Chronic pulmonary disease, no (%) | 14(6.4) | 7(26.9) | <0.001 |
| Asthma, no (%) | 43(19.6) | 3(11.5) | 0.32 |
| Renal, no (%) | 16(7.3) | 4(15.4) | 0.16 |
| Chronic neurological disease, no (%) | 10(4.6) | 2(7.7) | 0.49 |
| Cancer, no (%) | 8(3.7) | 3(11.5) | 0.066 |
| Diabetes, no (%) | 51(23.3) | 7(26.9) | 0.68 |
| Comorbidities^†^ | 165(75.3) | 20(76.9) | 0.072 |
| Hemoglobin, g/dL | 13.0±1.7 | 12.1±1.8 | 0.005 |
| WBC, *10^9/L | 6.9±3.5 | 6.4±3.4 | 0.26 |
| Lymphocytes*10^9^/L | 1.1±0.6 | 0.6±0.4 | <0.001 |
| Neutrophils*10^9^/L | 5.3±3.4 | 5.3±3.0 | 0.50 |
| Platelets, *10^9^/L | 229.3±88.3 | 183.3±70.5 | 0.006 |
| Creatinine, µmol/L | 72(58, 85) | 82(69, 106) | 0.062 |
| CRP mg/L | 52(24, 117) | 86(54, 142) | 0.040 |
| Ferritin µg/L | 583(269, 1027) | 878(527, 1593) | 0.013 |

Continuous data are given as mean±SD or median (25th, 75th) percentile. BMI, body mass index; CRP, c-reactive protein; ICU, intensive care unit; WBC, white blood cells. Comorbidities^†^ represents accumulated comorbidities + obesity.
