# Supplementary material for: Platelet-derived mediators in hospitalized COVID-19 patients and associations to respiratory failure, ICU admittance and 60-day mortality
Source: Front Cardiovasc Med. 2026 Feb 25;13:1685861. doi: 10.3389/fcvm.2026.1685861 (PMC12976018; doi:10.3389/fcvm.2026.1685861)
Supplement: Supplementary file 3 [file Table2.docx]

Supplementary table 2

Temporal profiles of platelet-derived mediators and platelet count during the acute phase in hospitalized COVID-19 patients according to use of dexamethasone and anticoagulants.

|  |  | Admission | 3-5 days | 7-10 days |  | p |
| --- | --- | --- | --- | --- | --- | --- |
| Dexamethasone | | |  |  |  |  |
| PF4  ng/mL | - | 2.58 [1.86-3.56] | 2.10 [1.49-2.96] | 2.88 [1.94-4.26] | grp | 0.92 |
|  | + | 2.84 [1.98-4.08] | 2.44 [1.71-3.49] | 2.32 [1.62-3.34] | grp×time | 0.06 |
| ENA-78  pg/mL | - | 166 [118-232] | 131 [90.1-191] | 184 [113-301] | grp | 0.013 |
|  | + | 121 [82.8-177]* | 101 [68.8-148] | 120 [80.4-180] | grp×time | 0.77 |
| VEGF-A  pg/mL | - | 143 [109-188] | 152 [114-204] | 168 [120-237] | grp | 0.33 |
|  | + | 131 [96.8-178] | 142 [104-193] | 145 [106-198] | grp×time | 0.85 |
| RANTES  pg/mL | - | 691 [582-820] | 628 [522-755] | 721 [572-909] | grp | 0.66 |
|  | + | 684 [565-827] | 659 [546-795] | 639 [526-777] | grp×time | 0.26 |
| P-selectin  ng/mL | - | 53.2 [45.1-62.7] | 50.2 [42.1-59.9] | 63.9 [51.0-80.0] | grp | 0.38 |
|  | + | 52.9 [44.0-63.6] | 59.1 [49.1-71.0]* | 64.5 [53.2-78.3] | grp×time | 0.014 |
| sCD40L  pg/mL | - | 221 [152-320] | 192 [130-283] | 304 [190-485] | grp | 0.53 |
|  | + | 274 [180-416] | 245 [162-370] | 250 [163-384] | grp×time | 0.046 |
| SDF-1  pg/mL | - | 97.8 [77.8-123] | 104 [82.0-132] | 119 [87.8-163] | grp | 0.025 |
|  | + | 111 [86.1-143] | 135 [105-173]** | 144 [111-188] | grp×time | 0.30 |
| NAP-2  ng/mL | - | 1.60 [1.20-2.14] | 1.49 [1.09-2.05] | 1.85 [1.26-2.71] | grp | 0.55 |
|  | + | 1.67 [1.21-2.31] | 1.55 [1.12-2.14] | 1.41 [1.01-1.96] | grp×time | 0.14 |
| Platelets  *10^9^/L | - | 180 [156-208] | 211 [181-245] | 299 [244-367] | grp | 0.79 |
|  | + | 169 [144-199] | 220 [187-259] | 291 [245-346] | grp×time | 0.046 |
| All anti-coagulants | | |  |  |  |  |
| PF4  ng/mL | - | 2.92 [1.90-4.50] | 2.30 [1.40-3.79] | 3.68 [2.15-6.28] | grp | 0.13 |
|  | + | 2.50 [1.86-3.35] | 2.09 [1.56-2.81] | 2.05 [1.51-2.79] | grp×time | 0.08 |
| ENA-78  pg/mL | - | 151 [96.0-236] | 110 [62.5-195] | 176 [88.4-349] | grp | 0.57 |
|  | + | 135 [99.3-185] | 112 [81.5-154] | 135 [95.6-191] | grp×time | 0.71 |
| VEGF-A  pg/mL | - | 157 [110-225] | 181 [117-278] | 170 [107-270] | grp | 0.07 |
|  | + | 119 [93.2-153] | 127 [98.9-164] | 133 [103-173] | grp×time | 0.84 |
| RANTES  pg/mL | - | 722 [573-909] | 627 [478-822] | 728 [528-1003] | grp | 0.48 |
|  | + | 667 [571-779] | 634 [542-741] | 631 [533-746] | grp×time | 0.56 |
| P-selectin  ng/mL | - | 54.9 [44.1-68.2] | 52.2 [40.3-67.7] | 69.8 [51.0-95.6] | grp | 0.81 |
|  | + | 52.7 [45.4-61.3] | 56.2 [48.3-65.4] | 62.8 [53.3-74.0] | grp×time | 0.38 |
| sCD40L  pg/mL | - | 244 [149-400] | 225 [131-389] | 320 [167-616] | grp | 0.60 |
|  | + | 248 [177-347] | 215 [154-302] | 236 [165-338] | grp×time | 0.50 |
| SDF-1  pg/mL | - | 97.5 [71.9-132] | 129 [91.4-181] | 160 [104-245] | grp | 0.74 |
|  | + | 108 [87.9-133] | 123 [100-151] | 132 [105-165] | grp×time | 0.28 |
| NAP-2  ng/mL | - | 1.88 [1.28-2.76] | 1.69 [1.06-2.69] | 2.30 [1.36-3.91] | grp | 0.022 |
|  | + | 1.42 [1.09-1.85] | 1.31 [1.01-1.71] | 1.23 [0.92-1.63] | grp×time | 0.27 |
| Platelets  *10^9^/L | - | 178 [148-214] | 175 [141-217] | 216 [161-289] | grp | 0.008 |
|  | + | 181 [159-206] | 234 [205-267]*** | 319 [275-369]** | grp×time | <0.001 |

Data shown are estimated marginal means and 95% CI adjusting for age, sex, platelet counts and comorbidity. Platelet counts were omitted as covariate when looking at effect on platelet counts. In addition, dexamethasone was included as covariate when assessing effects of anticoagulation and anti-coagulation was included when assessing effects of dexamethasone.

p-values in the right column reflect the group (i.e. categorized dexamethasone or anticoagulant treatment) effect, and group×time from the linear mixed models analysis. *p < 0.05, **p<0.01, ***p<0.001 between groups are sequential Sidak adjusted p-values from the mixed model analysis. Number of observations at each time point were dexamethasone -/+: Admission 97/129; 3-5 days 51/100; 7-10 days 20/74, anticoagulant treatment -/+: Admission 29/197; 3-5 days 12/139; 7-10 days 8/86.
