# Supplementary material for: Platelet-derived mediators in hospitalized COVID-19 patients and associations to respiratory failure, ICU admittance and 60-day mortality
Source: Front Cardiovasc Med. 2026 Feb 25;13:1685861. doi: 10.3389/fcvm.2026.1685861 (PMC12976018; doi:10.3389/fcvm.2026.1685861)
Supplement: Supplementary file 4 [file Table3.docx]

Supplementary Table 3

Demographic and clinical admission values in patients with or without follow-up samples.

|  | No follow-up  n = 114 | Follow-up  n = 176 | p |
| --- | --- | --- | --- |
| Age, years | 59.1±16.3 | 55.8±13.8 | 0.032 |
| Male sex, no (%) | 70 (61.4) | 106 (60.2) | 0.84 |
| BMI, kg/m2 | 28.5±5.2 | 29.1±4.6 | 0.20 |
| Obesity, no (%) | 38 (33.3) | 62 (35.2) | 0.74 |
| Symptom duration, days | 9.5±6.0 | 7.1±4.6 | <0.001 |
| Oxygen therapy, days | 7 (3,12) | 7 (3,11) | 0.71 |
| Dexamethasone, no (%) | 67 (58.8) | 84 (47.7) | 0.066 |
| Anticoagulants total, no (%) | 100 (87.7) | 150 (85.2) | 0.55 |
| Cardiovascular disease, no (%) | 22 (19.3) | 28 (15.9) | 0.46 |
| Hypertension, no (%) | 39 (34.2) | 62 (37.6) | 0.57 |
| Chronic pulmonary disease, no (%) | 11 (9.6) | 12 (6.8) | 0.38 |
| Asthma, no (%) | 17 (14.9) | 35 (19.9) | 0.28 |
| Renal, no (%) | 15 (13.2) | 10 (5.7) | 0.027 |
| Chronic neurological disease, no (%) | 9 (7.9) | 3 (1.7) | 0.010 |
| Cancer, no (%) | 5 (4.4) | 7 (4.0) | 0.86 |
| Diabetes, no (%) | 31 (27.2) | 35 (20.6) | 0.20 |
| Comorbidities^†^ | 85 (74.6) | 130 (73.9) | 0.90 |
| Hemoglobin, g/dL | 12.8±1.8 | 13.2±1.6 | 0.018 |
| WBC, *10^9/L | 6.4±3.0 | 7.7±8.9 | 0.072 |
| Lymphocytes*10^9^/L | 1.0±0.5 | 1.1±0.6 | 0.029 |
| Neutrophils*10^9^/L | 4.9±2.7 | 5.5±3.7 | 0.084 |
| Platelets, *10^9^/L | 215±80 | 233±101 | 0.056 |
| Creatinine, µmol/L | 71 (58, 87) | 74 (60, 86) | 0.53 |
| CRP mg/L | 53 (29, 115) | 64 (23, 125) | 0.96 |
| Ferritin µg/L | 590 (291, 881) | 608 (290, 1164) | 0.38 |

Continuous data are given as mean±SD or median (25th, 75th) percentile. BMI, body mass index; CRP, c-reactive protein; ICU, intensive care unit; WBC, white blood cells. Comorbidities^†^ represents accumulated comorbidities + obesity.
