# Supplementary material for: Platelet-derived mediators in hospitalized COVID-19 patients and associations to respiratory failure, ICU admittance and 60-day mortality
Source: Front Cardiovasc Med. 2026 Feb 25;13:1685861. doi: 10.3389/fcvm.2026.1685861 (PMC12976018; doi:10.3389/fcvm.2026.1685861)
Supplement: Supplementary file 5 [file Table4.docx]

Supplementary Table 4

Temporal profiles of platelet-related mediators and platelet count during the acute phase (A) and follow-up (B) in hospitalized COVID-19 patients according to sex.

| (A) |  | Admission | 3-5 days | 7-10 days | Mixed model | p |
| --- | --- | --- | --- | --- | --- | --- |
| Platelets | women | 186 [159-217] | 218 [187-255] | 265 [223-316] | grp | 0.559 |
| *10^9^/L | men | 172 [150-197] | 210 [183-241] | 274 [235-321] | grp×time | 0.202 |
| RANTES | women | 697 [571-849] | 666 [546-813] | 695 [566-854] | grp | 0.668 |
| pg/mL | men | 702 [588-839] | 663 [554-793] | 644 [535-776] | grp×time | 0.451 |
| P-selectin | women | 54 [45-64] | 55 [45-65] | 62 [51-75] | grp | 0.269 |
| pg/mL | men | 57 [49-67] | 60 [51-71] | 63 [53-75] | grp×time | 0.531 |
| sCD40L | women | 265 [177-399] | 242 [161-364] | 289 [190-441] | grp | 0.704 |
| pg/mL | men | 277 [193-400] | 241 [167-347] | 241 [165-353] | grp×time | 0.153 |
| SDF-1 | women | 99 [78-127] | 110 [86-140] | 118 [91-153] | grp | 0.001 |
| pg/mL | men | 128 [103-161]** | 142 [114-177]*** | 144 [114-182]* | grp×time | 0.744 |
| NAP-2 | women | 1.62 [1.13-2.31] | 1.62 [1.13-2.33] | 1.74 [1.2-2.51] | grp | 0.378 |
| ng/mL | men | 1.68 [1.22-2.32] | 1.5 [1.09-2.08] | 1.36 [0.98-1.89] | grp×time | 0.031 |
| PF4 | women | 2.85 [1.95-4.16] | 2.59 [1.77-3.77] | 2.76 [1.89-4.04] | grp | 0.176 |
| ng/mL | men | 2.77 [1.97-3.89] | 2.2 [1.57-3.09] | 2.15 [1.52-3.03] | grp×time | 0.132 |
| ENA-78 | women | 168 [114-247] | 153 [103-227] | 195 [129-295] | grp | <0.001 |
| pg/mL | men | 130 [92-184] | 102 [71-145]* | 110 [76-159]** | grp×time | 0.053 |
| VEGF-A | women | 142 [106-190] | 153 [113-207] | 155 [114-210] | grp | 0.725 |
| pg/mL | men | 142 [109-184] | 146 [112-191] | 147 [112-194] | grp×time | 0.698 |

| (B) |  | 3 months | 6 months | 12 months |
| --- | --- | --- | --- | --- |
| RANTES  pg/mL | women  men | 867 [769-975]  875 [796-959] | 693 [519-925]  684 [536-873] | 942 [838-1062]  944 [851-1045] |
| P-selectin pg/mL | women  men | 52 [47-58]  55 [50-59] | 58 [50-67]  66 [59-75] | 56 [50-63]  57 [51-63] |
| sCD40L  pg/mL | women  men | 308 [228-415]  286 [226-363] | 356 [237-537]  337 [239-476] | 372 [235-590]  308 [207-461] |
| SDF-1  pg/mL | women  men | 70 [62-79]  77 [70-85] | 72 [62-83]  74 [65-83] | 66 [56-77]  80 [70-92] |
| NAP-2  ng/mL | women  men | 1.3 [1.04-1.63]  1.17 [0.98-1.4] | 1.32 [0.87-2.01]  1.09 [0.76-1.54] | 1.59 [1.23-2.04]  1.31 [1.05-1.63] |
| PF4  ng/mL | women  men | 2.03 [1.6-2.58]  2.12 [1.76-2.56] | 2.47 [1.67-3.64]  2.43 [1.75-3.37] | 2.87 [2.18-3.78]  2.34 [1.84-2.97] |
| ENA-78  pg/mL | women  men | 349 [259-471]  234 [185-297]* | 266 [157-452]  202 [130-316] | 421 [295-600]  274 [201-373] |
| VEGF-A  pg/mL | women  men | 115 [100-134]  100 [89-112] | 128 [97-168]  106 [85-134] | 122 [102-148]  105 [89-124] |

Data shown are estimated marginal means and 95% CI adjusting for age and comorbidity. For acute phase samples in Table A, differences in temporal profiles were evaluated by linear mixed models. p-values in the right column reflect the sex effect, and sex×time from the linear mixed model analysis. For long-term data in B, differences between men and women were assessed by a multivariate general linear model at each time-point. *p < 0.05, **p<0.01, ***p<0.001 between men and women.
